# Community-Acquired Pneumonia among Patients with COPD in Spain from 2016 to 2019. Cohort Study Assessing Sex Differences in the Incidence and Outcomes Using Hospital Discharge Data

**DOI:** 10.3390/jcm10214889

**Published:** 2021-10-23

**Authors:** Javier de Miguel-Diez, Marta Lopez-Herranz, Valentin Hernandez-Barrera, Jose M. de Miguel-Yanes, Napoleon Perez-Farinos, Julia Wärnberg, David Carabantes-Alarcon, Rodrigo Jimenez-Garcia, Ana Lopez-de-Andres

**Affiliations:** 1 Respiratory Department, Instituto de Investigación Sanitaria Gregorio Marañón (IiSGM), Hospital General Gregorio Marañón, Universidad Complutense de Madrid, 28040 Madrid, Spain; javier.miguel@salud.madrid.org; 2 Nursing Department, Faculty of Nursing, Physiotherapy and Podology, Universidad Complutense de Madrid, 28040 Madrid, Spain; 3 Preventive Medicine and Public Health Teaching and Research Unit, Health Sciences Faculty, Universidad Rey Juan Carlos, Alcorcón, 28922 Madrid, Spain; valetin.hernandez@urjc.es; 4 Internal Medicine Department, Instituto de Investigación Sanitaria Gregorio Marañón (IiSGM), Hospital General Gregorio Marañón, Universidad Complutense de Madrid, 28040 Madrid, Spain; josemaria.demiguel@salud.madrid.org; 5 Instituto de Investigación Biomédica de Málaga (IBIMA), School of Medicine, Universidad de Málaga, 29071 Málaga, Spain; napoleon.perez@uma.es; 6 Instituto de Investigación Biomédica de Málaga (IBIMA), School of Health Sciences, Universidad de Málaga, 29071 Málaga, Spain; jwarnberg@uma.es; 7 Department of Public Health & Maternal and Child Health, Faculty of Medicine, Universidad Complutense de Madrid, 28040 Madrid, Spain; dcaraban@ucm.es (D.C.-A.); rodrijim@ucm.es (R.J.-G.); anailo04@ucm.es (A.L.-d.-A.)

**Keywords:** community-acquired pneumonia, COPD, sex, incidence, in-hospital mortality

## Abstract

Background: To describe and analyze the incidence and hospital outcomes of patients admitted with community-acquired pneumonia (CAP) according to Chronic Obstructive Pulmonary Disease (COPD) status and sex in Spanish hospitals from 2016 to 2019. Methods: We conducted a cohort study using national hospital discharge data of all patients ≥40 years with CAP. Results: A total of 500,833 patients (59.0% men) was identified. Incidence of CAP increased over time. Age-adjusted incidence was 4.42-times higher in COPD patients. In-hospital mortality (IHM) was lower in men and women with COPD than in those without COPD (14.41% vs. 10.70% in men; 11.12% vs. 8.58%. in women; *p* < 0.001). The risk of dying in hospital increased with age, presence of several comorbidities (excluding T2DM that was a protective factor), and need for mechanical ventilation (non-invasive and invasive) during admission, irrespective of sex. Over time, the IHM decreased significantly in men and women with COPD. Men with COPD were significantly more likely to die in hospital than were COPD women (OR 1.13; 95% CI 1.07–1.21). Conclusions: Incidence of CAP was higher among subjects with COPD, although the effect of COPD was higher in men than in women. By contrast, IHM was lower in COPD patients, but men with COPD were significantly more likely to die in hospital than were COPD women.

## 1. Introduction

Community acquired pneumonia (CAP) is an infection of the pulmonary parenchyma that results in a huge economic and health burden [1]. It is a major cause of hospitalizations, morbidity, and mortality [2]. Chronic obstructive pulmonary disease (COPD) increases the susceptibility to CAP [3,4]. COPD is the third leading cause of death worldwide [5]. COPD patients are at increased risk of developing pneumonia [6], which can be related to more pathogen colonization as a consequence of altered immune mechanisms, which results in higher susceptibility for infection [7]. In addition, COPD can be an adverse prognostic factor for patients hospitalized with pneumonia [8]. However, the influence of COPD on mortality related to CAP is inconclusive. Thus, some authors demonstrated that COPD reduced [9], increased [10], or did not affect mortality [11].

It has been previously reported that sex is a variable associated with the incidence and outcomes of CAP [12]. In fact, CAP has been described to be more severe in men than in women, leading to higher mortality in the former, especially in older age [12]. However, its effect in patients with CAP according to COPD status is unknown. Identifying sex differences in CAP patients with and without COPD can be useful to guide prognostication of patients and their management.

Large population-based hospital discharge databases can be useful to obtain a “real-world” perspective of the influence of COPD on CAP and to assess sex-differences in this possible association [13].

The objectives of this investigation are (1) to assess if suffering COPD affects the incidence of CAP in the Spanish population from 2016 to 2019; (2) to compare the clinical profile (comorbidities and procedures) and the mortality during the hospitalization between those with and without COPD and for women and men separately; and (3) to identify which of the study variables are independently associated with in-hospital mortality (IHM) for patients hospitalized with CAP according to sex and the presence of COPD.

## 2. Materials and Methods

A cohort study was carried out based on hospital discharge reports collected through the Hospital Discharge Records of the Spanish National Health System (HDRSNHS). According to the Spanish legislation, it is mandatory that all public and private hospitals send data on their discharges to the Ministry of Health every month. The study period was from 1 January 2016 to 31 December 2019. The discharge records are coded based on the International Classification of Disease, Tenth Revision (ICD-10). Details on HDRSNHS are available online [14].

The inclusion criteria were (1) patients aged ≥ 40 years; (2) patients discharged with a primary diagnosis of CAP, using the specific ICD-10 codes described in Table S1, and with a “Present on Admission” (POA) indicator of “Yes”, and (3) patients discharged with a secondary diagnosis of CAP (codes shown in Table S1) and with the POA indicator of “Yes”. We excluded those patients with “*Not specified sex*” and those lacking information regarding their length of hospital stay. The flowchart of the study sample selection can be found in Figure S1.

The population was divided according to sex and to COPD status. Subjects with a diagnosis code for COPD (J44.0, J44.1, J44.9) in any diagnosis field were classified as having COPD.

The main study variables were trends from the year 2016 to 2019 in the incidence of CAP, the length of hospital stay (LOHS), and the IHM among women and men with and without COPD. We also analyzed comorbidities and therapeutic procedures.

To estimate the incidences for the years analyzed, we obtained data from the Spanish National Institute of Statistics (SNIS) [15] and the Spanish National Health Survey for the year 2017 (SNHS2017) [16]. Combining these two databases, we calculated the number of women and men aged 40 years or over who suffered COPD in each of the years analyzed. These subpopulations were used as denominators to provide the yearly incidences.

Charlson Comorbidity Index (CCI) was calculated for each patient. This calculation was based on the ICD-10 codes in diagnosis positions from 1 to 20 and applying the algorisms proposed by Sundararajan et al. [17]. We described the prevalence of each single condition included in the CCI and of asthma and pulmonary tuberculosis (See ICD10 codes in Table S1).

The procedures analyzed included mechanical ventilation (non-invasive and invasive) (ICD-10 codes are shown Table S1).

### 2.1. Statistical Analysis

Incidences were analyzed with Poisson regression models. The measure of association obtained are incidence rate ratios (IRR) along with 95% confidence intervals (95% CI).

Descriptive statistical analysis included mean and standard deviation (SD) or median and interquartile range (IQR) for continuous variables, and absolute frequencies and percentages for categorical variables.

Continuous variables were compared using the *t*-test or Mann–Whitney test. Categorical variables were compared using the chi-square test.

We used the Propensity Score Matching (PSM) method to create subpopulations that were comparable based on the distribution of their baseline conditions [18]. We performed three PSM analyses, namely COPD men matched with non-COPD men, COPD women matched with non-COPD women, and COPD men matched with COPD women. The propensity score for each patient, required to do the PSM, was obtained with multivariable logistic regression [18]. In these multivariable models, we included age and all those chronic conditions studied that patients suffered when they were admitted to the hospital. However, due to the important differences in the prevalence of some concomitant conditions between sexes and in order to improve the matching using PSM for COPD men with COPD women, we had to include in the model only those chronic conditions with a prevalence over 5% in both sexes.

Study subpopulations obtained with PSM were compared with paired *t*-test or McNemar’s test as required [18].

To assess which of the study variables independently predicted IHM, we conducted multivariable logistic regression. Model construction was done following the steps proposed by Hosmer et al. [19].

The data depuration, statistical analysis, and PSM were done with Stata version 14 software (Stata, College Station, TX, USA).

### 2.2. Ethics

The HDRSNHS is owned by the Spanish Ministry of Health and provided free of charge upon request [20]. Given the characteristics of this registry, which is anonymous, it does not require individual written consent from the patients or ethics committee approval according to the Spanish legislation.

## 3. Results

A total of 500,833 patients aged ≥ 40 years (59.0% men and 41.0% women) were hospitalized in Spain with CAP in the period 2016–2019. COPD was codified in 95,546 patients (19.1%). The prevalence of COPD was 26.79% among men and 7.9% among women (*p* < 0.001).

### 3.1. Incidence of CAP Hospitalizations According to COPD Status

As can been seen in Table 1, among patients with COPD, we found that the incidence of CAP coding increased significantly from 153.86 in 2016 to 210.71 in 2019 cases per 100,000 persons with COPD (*p* < 0.001). This increment over the study period was also found in patients without COPD (*p* < 0.001). For every year analyzed, the incidence of CAP among COPD subjects was significantly higher than in the population without this condition (*p* < 0.001). After age-and-sex adjusted Poisson regression, the incidence of CAP was 4.42-times higher among the COPD population (IRR 4.42; 95% CI 4.39–4.45).

The incidence of CAP coding among those with COPD increased from 251.52 cases per 100,000 COPD men population and 51.06 cases per 100,000 COPD women population in 2016 to 353.44 and 74.44 in 2019, respectively (all *p* < 0.001). A similar trend was found among women and men without COPD (Table 1).

Age remained stable over time among those with and without COPD, with slightly higher age among COPD subjects (~77 vs. ~6 years). The CCI increased significantly over time in both patients with and without COPD (all *p* < 0.001). LOHS was around 7 days for all years analyzed and in addition to COPD status. IHM remained around 10% in patients with COPD over time but decreased significantly for patients without COPD from 13.78% in 2016 to 12.75% in 2019 (*p* < 0.001) (Table 1).

### 3.2. Clinical Characteristics and Hospital Outcomes for Women and Men with CAP According to COPD Status

Among women, as can be seen in Table 2, the incidence was significantly higher for those with COPD than in those without COPD (62.49 cases per 100,000 women with COPD vs. 36.30 cases per 100,000 women without COPD, *p* < 0.001), resulting in an adjusted IRR of 1.89 (95% CI 1.69–2.08).

Before PSM, the mean age was significantly lower among women with COPD (74.77; SD = 12.65 years) than non-COPD women (77.98; SD = 13.68 years), and also the mean CCI was lower. Non-COPD women had higher frequency of dementia (12.28% vs: 6.22%; *p* < 0.001), cerebrovascular disease, cancer, and renal disease (Table 2). Women with COPD had a higher prevalence of asthma (10.62% vs. 9.99%: *p* = 0.01).

Women with COPD received mechanical ventilation significantly more often than women without COPD (non-invasive: 5.28% vs. 2.48%; invasive: 2.74% vs. 1.92%; all *p* < 0.001). The mean LOHS was approximately 7 days in women with and without COPD. The crude IHM was 8.58% for women with COPD and 12.95% for non-COPD women (*p* < 0.001).

After PSM, non-invasive mechanical ventilation continued to be more frequent among COPD women; however, invasive ventilation was similar except for COPD status. IHM continued to be lower in non-COPD than in COPD women (8.58% vs. 11.12%: *p* < 0.001).

The results for men appear in Table 3. The crude incidence of CAP was significantly higher in men with COPD than in non-COPD men (298.34 cases per 100,000 men with COPD vs. 45.74 cases per 100,000 men without COPD; *p* < 0.001). The adjusted IRR was 5.04 (95% CI 4.93–5.16).

Before PSM, significant differences in the distribution age, and comorbidities between men with and without COPD were found. Men with COPD were older (77.44 years vs. 74.05 years) and had higher mean CCI (1.34 vs. 1.26) and higher prevalence of myocardial infarction, T2DM, congestive heart failure, renal disease, peripheral vascular disease, peptic ulcer, and mild liver disease. However, those without COPD had a higher prevalence of dementia, cerebrovascular disease, and cancer. Men with COPD received more non-invasive mechanical ventilation (4.25% vs. 2.51%; *p* < 0.001) but received less invasive mechanical ventilation (2.29% vs. 3.13%; *p* < 0.001). LOHS was around 7 days in both men with and without COPD. Non-COPD men had higher crude IHM than men with COPD (13.69% vs. 10.7%, *p* < 0.001).

After PSM, we found that among COPD men, non-invasive mechanical ventilation continued to be more frequent, and invasive ventilation continued to be less frequent. After PSM, the IHM continued to be higher in non-COPD men (14.41% vs. 10.70%; *p* < 0.001).

### 3.3. Sex Differences for COPD Patients Hospitalized with CAP

As can be seen in Table 4, the incidence was significantly lower in women than in men with COPD. The overall age-adjusted incidence of CAP over the period 2016–2019 was 4.77 times higher among men with COPD than among women with COPD (IRR 4.77; 95% CI 4.69–4.85 according to the Poisson regression analysis). The IRR among those without COPD was 1.26 (95% 1.25–1.26).

Men with COPD were older (77.44 ± 10.21 years vs. 74.77 ± 12.65 years; *p* < 0.001) and with a higher mean CCI (1.34 ± 1.13 vs. 1.06 ± 1.04) than COPD women. Men more frequently had a code for myocardial infarction, T2DM, cerebrovascular disease, peripheral vascular disease, liver disease (mild and moderate/severe), renal disease, cancer, and metastatic cancer.

However, congestive heart failure, dementia, rheumatoid disease, peptic ulcer, and AIDS were more prevalent in women than in men. The prevalence of asthma was over three times higher among women with COPD than men with COPD (10.62% vs. 3.04; *p* < 0.001).

The difference in IHM was statistically significant (*p* < 0.001) before and after PSM, with proportions of 9.61% for men with COPD and 8.58% for women with COPD after PSM.

### 3.4. Variables Associated with Dying during Hospitalization among Women and Men with CAP According to COPD Status

As can been seen in Table 5, the IHM increased with age and with the suffering of congestive heart failure, renal disease, cancer or metastatic cancer, cerebrovascular disease, moderate or severe liver disease, hemiplegia or paraplegia, and dementia, among men and women with COPD. Myocardial infarction was only a factor associated with IHM in men, but not in women, with COPD (OR 1.18; 95% CI 1.08–1.28).

In both men and women with COPD, the presence of T2DM reduced the IHM (OR 0.85%; 95% CI 0.81–0.89 and OR 0.83; 95% CI 0.73–0.95, respectively). Among women with COPD, the presence of asthma reduced the risk of dying in the hospital (OR 0.74; 95% CI 0.62–0.89).

The need for mechanical ventilation (non-invasive and invasive) during admission increased the risk of IHM in COPD patients irrespective of sex (OR 2.56; 95% CI 2.36–2.79 and OR 8.71; 95% CI 7.9–9.6, respectively).

Over time, the IHM decreased significantly in both men and women with COPD. Finally, as found with the PSM, men with COPD were significantly more likely to die in hospital than were COPD women (OR 1.13; 95% CI 1.07–1.21).

In Table S2 are shown the variables associated with IHM among the non-COPD population according to sex. The variables that increased and reduced the risk of dying were the same described for the men and women with COPD. However, among non-COPD men, the risk of dying increased among those suffering from AIDS and decreased if a code for asthma was recorded. These two significant associations were not found among men with COPD

As reported for those with COPD, the IHM was higher among men than women, and the IHM decreased significantly from 2016 to 2019 for both sexes.

## 4. Discussion

In this nationwide population-based cohort study, we found that the incidence of CAP increased significantly from 2016 to 2019, both in COPD and non-COPD patients. It is important to note that ICD-10 was implemented in Spain as a reference classification for clinical coding from 1 January 2016, and it is possible that coding may became more accurate over time. However, these results are in agreement with studies carried out in periods priors to ours, and they did not appear to be fully explained by demographic changes or by an increase in co-existing conditions [21,22,23]. In addition, we reported that after multivariable adjustment in the population with COPD, the incidence of CAP was four times higher than in the population without COPD. In previous studies, prior COPD exacerbation, COPD severity, low body mass index, more comorbid conditions, use of inhaled corticosteroid (ICS), smoking habit, suboptimal immunization, and higher age have been identified as risk factors for CAP in persons suffering from COPD [4,13,24].

By sex, the incidence of CAP increased in both men and women with and without COPD, although it was also significantly higher in men and women with COPD than in those without COPD, especially among men. Other studies have also shown that the incidence of CAP is higher in men than in women [3,25], but there are no previous studies in the literature that assess the influence of sex on the relationship between CAP and COPD. In any case, it is possible that biological, cultural, and behavioral factors, as well as socioeconomic differences, could partly justify these results in patients with CAP and COPD [12].

We agree with Cavallazzi et al., finding that among CAP, those with concomitant COPD suffer more comorbidities and are older [26]. In this regard, we observed that the mean age and the mean CCI increased significantly over time in addition to COPD status. Among men, we did not detect significant differences in mean age between those with and without COPD, but comorbidity was slightly higher in COPD patients. Among women, we did not detect significant differences after matching in age or comorbidity between those with and without COPD. Furthermore, we observed that men with CAP and COPD had a higher mean CCI than women with both conditions. In the same way, Alsawas et al. [27] analyzed the sex disparities between hospitalized patients with pneumonia and found that the prevalence of comorbidities was significantly different between men and women, such that men had multi-comorbidities more frequently than women. In the same study, multi-comorbidities had a significant effect on readmission at 30 days in men, but not in women.

Despite the increase in comorbidity, IHM decreased significantly from year 2016 to year 2019 in COPD in women and men, suggesting an improvement in the clinical management of COPD over time. A remarkable result is that the IHM was lower in women and men who suffered COPD hospitalized with CAP than in those without this chronic disease.

In the studies published to date, it is still unclear whether COPD is associated with a better or worse survival after CAP [9,10,11]. The opposite conclusions found in these studies may be due to differences in the study design, sample population characteristics regarding age and sex, prevalence of smoking habit, COPD severity, associated comorbidities, established treatment, and use of healthcare resources [28]. However, the lower mortality among COPD patients has been reported by Dusemund et al., who conducted an age–sex matched case-control study in Switzerland from 2002 to 2010 analyzing 17.075 patients [29]. Furthermore, two national population-based cohort studies conducted in Germany and Spain reported COPD as a protective factor for mortality after CAP [9,23]. The lower-than-expected mortality among COPD patients could be due to multiple reasons including biological factors such as a different immune response or a beneficial anti-inflammatory effect caused by inhaled corticosteroids [26,29]. Other explanation could be the increased awareness of patients with COPD and clinicians treating patients with this condition that would result in an earlier diagnosis, treatment, and hospital admission with a less severe CAP. Despite the lack of a damaging short-term effect of COPD, it is possible that COPD patients have a worse mid and long-term mortality after CAP, as recently described by Bordon et al. [24]. As reported by other authors [11,30,31], after multivariable adjustment, the short-term mortality did not differ among COPD patients. Notwithstanding these findings, some studies detected an association between COPD and mortality in patients with CAP [8,10,32], and in others, this association became not significant when other risk factors were included in the model [33].

In the future large prospective studies should be done to clarify the role of COPD on the outcome of CAP [2].

On the other hand, we found that men with COPD were significantly more likely to die in hospital than were COPD women. Several authors have also shown that CAP is more severe and results in higher mortality among men than women, especially in older age [12,34]. Sex-differences might be due to biologic differences [12] but also to many other factors, including access to care [35].

To our knowledge, few studies have assessed risk factors of mortality after CAP in patients hospitalized with concomitant COPD [36]. We identified that the risk factors for mortality among men and women with COPD were older age, suffering congestive heart failure, renal disease, cancer or metastatic cancer, cerebrovascular disease, moderate or severe liver disease, hemiplegia or paraplegia, and dementia. However, the presence of T2DM reduced the IHM in both groups. T2DM is strongly associated with high body mass index, and the existence of an obesity paradox has been suggested for CAP [37]. The obesity paradox refers to epidemiological evidence showing that obesity in patients may have a protective effect that leads to decreased mortality among hospitalized patients [37].

Among women with COPD, the presence of concomitant asthma was associated with lower IHM after CAP. In a previous study, Yamauchi et al. also demonstrated that in-hospital mortality was higher in patients with COPD alone than in those with ACOS [38]. Both asthma and COPD patients are ICS users and, as previously described, association between asthma/COPD and lower IHM may be due to selection bias [39]. However, the association persisted even after taking into account known covariates by applying multivariate regression analysis. Another possible explanation for the protective effect of asthma on the IHM may be that patients with this concomitant condition are admitted with less severe CAP due to the asthmatic symptomatology, but this is unclear.

As in other studies [40], the need for mechanical ventilation during admission also increased the risk of IHM in COPD–CAP patients irrespective of sex.

Our study has strengths and limitations. The strengths include the population-based design and the large sample size, covering the entire population of Spain. The main limitation is related to use of an administrative database. Therefore, we lacked information on clinical variables, such as severity of pneumonia, degree of airway obstruction, antibiotic therapy, use of corticosteroids, and history of influenza or pneumococcal immunizations. In addition, even if our database had high specificity, is possible that some conditions and procedures were under-reported [41]. Furthermore, in our investigation we could not analyze the asthma–COPD overlap syndrome (ACOS) because, unfortunately, there is no specific code for ACOS in the ICD10 [42].

Finally, we did not evaluate the influence of COPD in CAP patients that did not require hospitalization.

## 5. Conclusions

In conclusion, we found that COPD increased the incidence of CAP, although its effect was higher in men than in women. IHM was lower in COPD patients of both sexes than in those without COPD. Men with COPD were significantly more likely to die in hospital than COPD women. Our results indicate that sex may be an important determinant of incidence and mortality of pneumonia in COPD patients, adding information to the existing literature in this field.

## Figures and Tables

**Table 1:** Trends in the incidence, age distribution, Charlson comorbidity index, and hospital outcomes for patients admitted to Spanish hospitals from the year 2016 to 2019 with community-acquired pneumonia according to presence of chronic obstructive pulmonary disease.

		2016	2017	2018	2019	*p*-Value
N, Incidence per 100,000 subjects	COPD	21,733 (153.86)	23,992 (169.85)	25,499 (198.68)	24,322 (210.71)	<0.001
	No COPD	87,962 (36.21)	97,936 (40.32)	111,577 (44.62)	107,812 (41.91)	<0.001
N, Incidence per 100,000 men	COPD	18,219 (251.52)	20,031 (276.54)	21,081 (327.31)	19,926 (353.44)	<0.001
	No COPD	47,574 (41.19)	52,055 (45.07)	59,261 (49.7)	57,618 (46.84)	<0.001
N, Incidence per 100,000 women	COPD	3514 (51.06)	3961 (57.56)	4418 (69.1)	4396 (74.44)	<0.001
	No COPD	40,388 (31.7)	45,881 (36.01)	52,316 (39.99)	50,194 (37.38)	<0.001
Age, mean (SD)	COPD	76.81 (10.69)	77.31 (10.51)	76.92 (10.79)	76.89 (10.85)	<0.057
	No COPD	75.24 (13.98)	76.28 (13.61)	76 (13.76)	75.91 (13.94)	<0.001
40–64, *n* (%)	COPD	2936 (13.51)	3069 (12.79)	3574 (14.02)	3505 (14.41)	<0.001
	No COPD	19,880 (22.6)	19,782 (20.2)	23,480 (21.04)	23,457 (21.76)	<0.001
65–74, *n* (%)	COPD	5011 (23.06)	5434 (22.65)	5943 (23.31)	5638 (23.18)	0.141
	No COPD	15,232 (17.32)	17,137 (17.5)	19,746 (17.7)	19,037 (17.66)	<0.001
75–84, *n* (%)	COPD	8168 (37.58)	8906 (37.12)	8956 (35.12)	8355 (34.35)	<0.001
	No COPD	26,164 (29.74)	28,540 (29.14)	31,524 (28.25)	29,041 (26.94)	<0.001
≥85, *n* (%)	COPD	5618 (25.85)	6583 (27.44)	7026 (27.55)	6824 (28.06)	<0.001
	No COPD	26,686 (30.34)	32,477 (33.16)	36,827 (33.01)	36,277 (33.65)	<0.001
CCI index, mean (SD)	COPD	1.22 (1.1)	1.27 (1.1)	1.3 (1.13)	1.35 (1.15)	<0.001
	No COPD	1.14 (1.06)	1.21 (1.08)	1.21 (1.09)	1.25 (1.11)	<0.001
LOHS, median (IQR)	COPD	7 (7)	7 (6)	7 (6)	7 (6)	0.231
	No COPD	7 (8)	7 (7)	7 (7)	7 (7)	0.076
IHM, *n* (%)	COPD	2279 (10.49)	2490 (10.38)	2696 (10.57)	2412 (9.92)	0.081
	No COPD	12,125 (13.78)	13,424 (13.71)	14,801 (13.27)	13,743 (12.75)	<0.001

CAP: community-acquired pneumonia; CCI: Charlson comorbidity index; COPD: chronic obstructive pulmonary disease; LOHS: length of hospital stay; IHM: in-hospital mortality.

**Table 2:** Incidence, age distribution, Charlson comorbidity index, concomitant diseases, and hospital outcomes for women admitted to Spanish hospitals from the year 2016 to 2019 with community-acquired pneumonia according to presence of chronic obstructive pulmonary disease. Results before and after propensity score matching.

	BEFORE PSM			AFTER PSM		
	COPD	No COPD	*p*-Value	COPD	No COPD	*p*-Value
N (incidence per 100,000 women)	16,289 (62.49)	188,779 (36.30)	<0.001			
Age, mean (SD)	74.77 (12.65)	77.98 (13.68)	<0.001	74.77 (12.65)	74.79 (12.71)	0.895
40–64, *n* (%)	3914 (24.03)	33,942 (17.98)	<0.001	3914 (24.03)	3919 (24.06)	0.948
50–64, *n* (%)	3608 (22.15)	26,970 (14.29)	<0.001	3608 (22.15)	3554 (21.82)	0.470
65–74, *n* (%)	4272 (26.23)	52,102 (27.6)	<0.001	4272 (26.23)	4295 (26.37)	0.772
≥85, *n* (%)	4495 (27.6)	75,765 (40.13)	<0.001	4495 (27.6)	4521 (27.75)	0.747
CCI index, mean (SD)	1.06 (1.04)	1.14 (1.05)	<0.001	1.06 (1.04)	1.05 (1.03)	0.512
Myocardial infarction, *n* (%)	524 (3.22)	5512 (2.92)	0.031	524 (3.22)	506 (3.11)	0.569
Congestive heart failure, *n* (%)	4422 (27.15)	49,947 (26.46)	0.056	4422 (27.15)	4414 (27.1)	0.921
Peripheral vascular disease, *n* (%)	529 (3.25)	4606 (2.44)	<0.001	529 (3.25)	510 (3.13)	0.549
Cerebrovascular disease, *n* (%)	687 (4.22)	11,334 (6)	<0.001	687 (4.22)	666 (4.09)	0.560
Dementia, *n* (%)	1013 (6.22)	23,191 (12.28)	<0.001	1013 (6.22)	1012 (6.21)	0.982
Rheumatoid disease, *n* (%)	552 (3.39)	7245 (3.84)	0.004	552 (3.39)	540 (3.32)	0.712
Peptic ulcer, *n* (%)	78 (0.48)	770 (0.41)	0.176	78 (0.48)	68 (0.42)	0.407
Mild liver disease, *n* (%)	869 (5.33)	6650 (3.52)	<0.001	869 (5.33)	859 (5.27)	0.805
T2DM, *n* (%)	4288 (26.32)	49,060 (25.99)	0.348	4288 (26.32)	4300 (26.4)	0.880
Hemiplegia or paraplegia, *n* (%)	82 (0.5)	1355 (0.72)	0.002	82 (0.5)	73 (0.45)	0.469
Renal disease, *n* (%)	2628 (16.13)	34,157 (18.09)	<0.001	2628 (16.13)	2624 (16.11)	0.952
Cancer, *n* (%)	799 (4.91)	11,805 (6.25)	<0.001	799 (4.91)	777 (4.77)	0.570
Moderate/severe liver disease, *n* (%)	120 (0.74)	1284 (0.68)	0.401	120 (0.74)	108 (0.66)	0.425
Metastatic cancer, *n* (%)	371 (2.28)	6167 (3.27)	<0.001	371 (2.28)	367 (2.25)	0.882
AIDS, *n* (%)	230 (1.41)	1077 (0.57)	<0.001	230 (1.41)	219 (1.34)	0.601
Asthma, *n* (%)	1737 (10.62)	19,748 (9.99)	0.010	1737 (10.62)	1632 (10.02)	0.105
Pulmonary tuberculosis, *n* (%)	6 (0.04)	165 (0.08)	0.08	6 (0.04)	20 (0.12)	0.006
Non-invasive mechanical ventilation, *n* (%)	860 (5.28)	4689 (2.48)	<0.001	860 (5.28)	358 (2.2)	<0.001
Invasive mechanical ventilation, *n* (%)	447 (2.74)	3620 (1.92)	<0.001	447 (2.74)	483 (2.97)	0.231
LOHS, median (IQR)	7 (6)	7 (7)	<0.001	7 (6)	7 (7)	0.324
IHM, *n* (%)	1398 (8.58)	24,454 (12.95)	<0.001	1398 (8.58)	1811 (11.12)	<0.001

PSM: propensity score matching; CAP: community-acquired pneumonia; COPD: chronic obstructive pulmonary disease; CCI: Charlson comorbidity index; T2DM: type 2 diabetes mellitus; AIDS: acquired immune deficiency syndrome; LOHS: length of hospital stay; IHM: in-hospital mortality.

**Table 3:** Incidence, age distribution, Charlson comorbidity index, concomitant diseases, and hospital outcomes for men admitted to Spanish hospitals from the year 2016 to 2019 with community-acquired pneumonia according to presence of chronic obstructive pulmonary disease. Results before and after propensity score matching.

	BEFORE PSM			AFTER PSM		
	COPD	No COPD	*p*-Value	COPD	No COPD	*p*-Value
N (incidence per 100,000 men)	79,257 (298.34)	216,508 (45.74)	<0.001			
Age, mean (SD)	77.44 (10.21)	74.05 (13.69)	<0.001	77.44 (10.21)	77.45 (10.21)	0.889
40–64, *n* (%)	9170 (11.57)	52,657 (24.32)	<0.001	9170 (11.57)	9284 (11.71)	0.372
50–64, *n* (%)	18,418 (23.24)	44,182 (20.41)	<0.001	18,418 (23.24)	18,351 (23.15)	0.690
65–74, *n* (%)	30,113 (37.99)	63,167 (29.18)	<0.001	30,113 (37.99)	29,962 (37.80)	0.434
≥85, *n* (%)	21,556 (27.2)	56,502 (26.1)	<0.001	21,556 (27.2)	21,660 (27.33)	0.344
CCI index, mean (SD)	1.34 (1.13)	1.26 (1.11)	<0.001	1.34 (1.13)	1.33 (1.1)	0.265
Myocardial infarction, *n* (%)	5426 (6.85)	13,828 (6.39)	<0.001	5426 (6.85)	5483 (6.92)	0.572
Congestive heart failure, *n* (%)	20,301 (25.61)	42,225 (19.5)	<0.001	20,301 (25.61)	20,278 (25.56)	0.894
Peripheral vascular disease, *n* (%)	7453 (9.4)	14,950 (6.91)	<0.001	7453 (9.4)	7336 (925)	0.312
Cerebrovascular disease, *n* (%)	4946 (6.24)	15,800 (7.3)	<0.001	4946 (6.24)	5001 (6.31)	0.569
Dementia, *n* (%)	4390 (5.54)	18,097 (8.36)	<0.001	4390 (5.54)	4506 (5.69)	0.205
Rheumatoid disease, *n* (%)	1484 (1.87)	4090 (1.89)	0.768	1484 (1.87)	1606 (2.03)	0.027
Peptic ulcer, *n* (%)	635 (0.8)	1456 (0.67)	<0.001	635 (0.8)	586 (0.79)	0.159
Mild liver disease, *n* (%)	4903 (6.19)	12,851 (5.94)	0.011	4903 (6.19)	4626 (5.83)	0.003
T2DM, *n* (%)	26,073 (32.9)	60,612 (28)	<0.001	26,073 (32.9)	25,782 (32.53)	0.119
Hemiplegia or paraplegia, *n* (%)	353 (0.45)	2232 (1.03)	<0.001	353 (0.45)	407 (0.51)	0.049
Renal disease, *n* (%)	17,108 (21.59)	41,701 (19.26)	<0.001	17,108 (21.59)	16,882 (21.35)	0.167
Cancer, *n* (%)	7776 (9.81)	24,040 (11.1)	<0.001	7776 (9.81)	7921 (9.99)	0.222
Moderate/severe liver disease, *n* (%)	796 (1)	3088 (1.43)	<0.001	796 (1)	699 (0.88)	0.011
Metastatic cancer, *n* (%)	3499 (4.41)	13,912 (6.43)	<0.001	3499 (4.41)	3611 (4.55)	0.174
AIDS, *n* (%)	632 (0.8)	2739 (1.27)	<0.001	632 (0.8)	665 (0.81)	0.933
Asthma, *n* (%)	2041 (3.04)	6565 (3.03)	0.675	2041 (3.04)	1932 (2.44)	0.079
Pulmonary tuberculosis, *n* (%)	83 (0.10)	326 (0.15)	0.003	83 (0.10)	94 (0.12)	0.408
Non-invasive mechanical ventilation, *n* (%)	3365 (4.25)	5444 (2.51)	<0.001	3365 (4.25)	1806 (2.41)	<0.001
Invasive mechanical ventilation, *n* (%)	1813 (2.29)	6778 (3.13)	<0.001	1813 (2.29)	2053 (2.59)	<0.001
LOHS, median (IQR)	7 (6)	7 (8)	0.063	7 (6)	7 (8)	0.0102
IHM, *n* (%)	8479 (10.7)	29639 (13.69)	<0.001	8479 (10.70)	11,424 (14.41)	<0.001

PSM: propensity score matching; CAP: community-acquired pneumonia; COPD: chronic obstructive pulmonary disease; CCI: Charlson comorbidity index; T2DM: type 2 diabetes mellitus; AIDS: acquired immune deficiency syndrome; LOHS: length of hospital stay; IHM: in-hospital mortality.

**Table 4:** Incidence, age distribution, Charlson comorbidity index, concomitant diseases and hospital outcomes for men and women suffering chronic obstructive pulmonary disease admitted to Spanish hospitals from year 2016 to 2019 with community-acquired pneumonia. Results before and after propensity score matching.

	BEFORE PSM			AFTER PSM		
	COPD MEN	COPD WOMEN	*p*-Value	COPD MEN	COPD WOMEN	*p*-Value
N (incidence per 100,000 subjects)	79,257 (298.34)	16,289 (62.49)				
Age, mean (SD)	77.44 (10.21)	74.77 (12.65)	<0.001	74.59 (11.95)	74.77 (12.64)	<0.062
40–64, *n* (%)	9170 (11.57)	3914 (24.03)	<0.0010	3889 (23.88)	3910 (24.01)	0.745
50–64, *n* (%)	18,418 (23.24)	3608 (22.15)	0.002	3585 (22.04)	3608 (22.16)	0.759
65–74, *n* (%)	30,113 (37.99)	4272 (26.23)	<0.001	4320 (26.53)	4272 (26.23)	0.546
≥85, *n* (%)	21,556 (27.2)	4495 (27.6)	0.299	4491 (27.58)	4495 (27.60)	0.960
CCI index, mean (SD)	1.34 (1.13)	1.06 (1.04)	<0.001	1.07 (1.06)	1.06 (1.04)	0.120
Myocardial infarction, *n* (%)	5426 (6.85)	524 (3.22)	<0.001	601 (3.69)	524 (3.22)	0.019
Congestive heart failure, *n* (%)	20,301 (25.61)	4422 (27.15)	<0.001	4400 (27.02)	4422 (27.15)	0.783
Peripheral vascular disease, *n* (%)	7453 (9.4)	529 (3.25)	<0.001	723 (4.44)	529 (3.25)	<0.001
Cerebrovascular disease, *n* (%)	4946 (6.24)	687 (4.22)	<0.001	708 (4.35)	687 (4.22)	0565
Dementia, *n* (%)	4390 (5.54)	1013 (6.22)	0.001	956 (5)	1013 (6.22)	0.185
Rheumatoid disease, *n* (%)	1484 (1.87)	552 (3.39)	<0.001	458 (2.81)	552 (3.39)	0.002
Peptic ulcer, *n* (%)	635 (0.8)	78 (0.48)	<0.001	97 (0.60)	78 (0.48)	0.150
Mild liver disease, *n* (%)	4903 (6.19)	869 (5.33)	<0.001	932 (5.72)	868 (5.33)	0.127
T2DM, *n* (%)	26,073 (32.9)	4288 (26.32)	<0.001	4317 (26.50)	4288 (26.33)	0.716
Hemiplegia or paraplegia, *n* (%)	353 (0.45)	82 (0.5)	0.316	60 (0.37)	82 (0.5)	0.064
Renal disease, *n* (%)	17,108 (21.59)	2628 (16.13)	<0.001	2673 (16.41)	2624 (16.11)	0.498
Cancer, *n* (%)	7776 (9.81)	799 (4.91)	<0.001	875 (5.373)	799 (4.91)	0.056
Moderate/severe liver disease, *n* (%)	796 (1)	120 (0.74)	0.001	156 (0.96)	120 (0.74)	0.030
Metastatic cancer, *n* (%)	3499 (4.41)	371 (2.28)	<0.001	446 (2.74)	371 (2.28)	0.008
AIDS, *n* (%)	632 (0.8)	230 (1.41)	<0.001	180 (1,11)	229 (1.41)	0.013
Asthma, *n* (%)	2041 (3.04)	1737 (10.62)	<0.001	1635 (10.04)	1737 (10.62)	0.065
Pulmonary tuberculosis, *n* (%)	83 (0.10)	6 (0.04)	0.013	16 (0.10)	6 (0.04)	0.050
Non-invasive mechanical ventilation, *n* (%)	3365 (4.25)	860 (5.28)	<0.001	711 (4.36)	860 (5.28)	<0.001
Invasive mechanical ventilation, *n* (%)	1813 (2.29)	447 (2.74)	<0.001	450 (2.76)	447 (2.74)	0.919
LOHS, median (IQR)	7 (6)	7 (6)	0.044	7 (6)	7 (6)	0.859
IHM, *n* (%)	8479 (10.7)	1398 (8.58)	<0.001	1566 (9.61)	1398 (8.58)	0.001

PSM: propensity score matching; CAP: community-acquired pneumonia; COPD: chronic obstructive pulmonary disease; CCI: Charlson comorbidity index; T2DM: type 2 diabetes mellitus; AIDS: acquired immune deficiency syndrome; LOHS: length of hospital stay; IHM: in-hospital mortality.

**Table 5:** Multivariable analysis to assess time trend and to identify study variables independently associated with in-hospital mortality among men and women hospitalized with CAP in Spain from 2016 to 2019.

	MEN	WOMEN	BOTH
	OR (95% CI)	OR (95% CI)	OR (95% CI)
40–64 years old, *n* (%)	1	1	1
50–64 years old, *n* (%)	1.40 (1.26–1.56)	1.89 (1.49–2.39)	1.51 (1.38–1.67)
65–74 years old, *n* (%)	2.14 (1.94–2.36)	3.39 (2.71–4.25)	2.35 (2.15–2.58)
≥85 years old, *n* (%)	3.54 (3.2–3.93)	6.91 (5.52–8.64)	4.03 (3.67–4.42)
Myocardial infarction	1.18 (1.08–1.28)	NS	1.17 (1.07–1.27)
Congestive heart failure	1.42 (1.35–1.49)	1.49 (1.32–1.69)	1.44 (1.37–1.51)
Cerebrovascular disease	1.54 (1.41–1.67)	1.63 (1.3–2.05)	1.55 (1.43–1.67)
Dementia	1.99 (1.83–2.16)	1.92 (1.61–2.3)	1.99 (1.84–2.14)
T2DM	0.85 (0.81–0.89)	0.84 (0.73–0.96)	0.85 (0.81–0.89)
Hemiplegia or paraplegia	2.71 (2.09–3.52)	3.55 (2.04–6.2)	2.83 (2.24–3.59)
Renal disease	1.23 (1.17–1.3)	1.19 (1.03–1.38)	1.23 (1.17–1.29)
Cancer	2.07 (1.93–2.22)	2.23 (1.77–2.81)	2.08 (1.95–2.22)
Moderate/severe liver disease	2.93 (2.43–3.52)	2.4 (1.42–4.06)	2.9 (2.44–3.45)
Metastatic cancer	5.32 (4.89–5.8)	8.95 (6.86–11.68)	5.6 (5.16–6.07)
Asthma	NS	0.74 (0.62–0.89)	0.78 (0.69–0.9)
Non-invasive mechanical ventilation	2.52 (2.3–2.77)	2.85 (2.33–3.49)	2.57 (2.36–2.79)
Invasive mechanical ventilation	8.65 (7.78–9.61)	10.35 (8.12–13.2)	8.82 (8.01–9.72)
2016	1	1	1
2017	0.98 (0.91–1.04)	0.89 (0.76–1.05)	0.96 (0.91–1.03)
2018	0.99 (0.93–1.06)	0.84 (0.72–0.99)	0.97 (0.91–1.03)
2019	0.91 (0.85–0.98)	0.71 (0.6–0.84)	0.88 (0.82–0.94)
Women	NA	NA	1
Men	NA	NA	1.12 (1.05–1.19)

T2DM: type 2 diabetes mellitus; NA: not applicable; NS: not significant. The absence of the condition or procedure is used as the reference category.

## Data Availability

According to the contract signed with the Spanish Ministry of Health and Social Services, which provided access to the databases from the Hospital Discharge Records of the Spanish National Health System (In Spanish: Conjunto Mínimo Basico de Datos (CMBD)), we cannot share the databases with any other investigator, and we have to destroy the databases once the investigation has concluded. Consequently, we cannot upload the databases to any public repository. However, any investigator can apply for access to the databases by filling out the questionnaire available at http://www.msssi.gob.es/estadEstudios/estadisticas/estadisticas/estMinisterio/SolicitudCMBDdocs/Formulario_Peticion_Datos_CMBD.pdf (accessed on 16 March 2021). All other relevant data are included in the paper.

## Supplementary Materials

The following is available online at https://www.mdpi.com/article/10.3390/jcm10214889/s1, Table S1: ICD-10 codes for diagnosis and therapeutic procedures. Table S2: Multivariable analysis of factors associated with in-hospital mortality during admissions for community-acquired pneumonia, among patients not suffering chronic obstructive pulmonary disease according to sex. Figure S1: Flow chart of patient selection and hospital outcome according COPD status and sex.
