# Supplementary material for: Community-Acquired Pneumonia among Patients with COPD in Spain from 2016 to 2019. Cohort Study Assessing Sex Differences in the Incidence and Outcomes Using Hospital Discharge Data
Source: J Clin Med. 2021 Oct 23;10(21):4889. doi: 10.3390/jcm10214889 (PMC8584564; doi:10.3390/jcm10214889)
Supplement: Supplementary file 1 [file jcm-10-04889-s001.zip › Tables S1 and S2.pdf]

Table S1. ICD-10 codes for diagnosis and therapeutic procedures used in this investigation.

	ICD-10 codes
Community-acquired pneumonia *	J12 to J18 as primary diagnosis with a POA indicator of "Y".<br>J12 to J18 in any of the secondary diagnosis fields (2-20) and with a POA indicator of "Y".
Asthma	J45.xxx
Pulmonary tuberculosis	A15.x
No invasive mechanical ventilation	5A09357, 5A09457, 5A09557
Invasive mechanical ventilation	5A1945Z, 5A1955Z, 5A1935Z

*Each discharge diagnosis has a "Present on Admission (POA)" indicator assigned according to the ICD-10-CM Official Guidelines for Coding and Reporting (https://icdlist.com/icd-10/guidelines/). The reporting options and definitions for POA are "Y" (present at admission); "N" (not present at admission); "U" (lack documentation to determine presence at admission); "W" (provider is unable to clinically determine if the condition was present); and unreported/not used.

Table S2. Multivariable analysis of factors associated with in-hospital mortality during admissions for community-acquired pneumonia, among patients not suffering chronic obstructive pulmonary disease according to sex.

	MEN	WOMEN	BOTH
	OR (95%CI)	OR (95%CI)	OR (95%CI)
40-64 years old, n (%)	1	1	1
50-64 years old, n (%)	1.44(1.38-1.51)	1.47(1.37-1.57)	1.45(1.4-1.51)
65-74 years old, n (%)	2.16(2.07-2.26)	2.73(2.57-2.9)	2.34(2.26-2.42)
≥ 85 years old, n (%)	3.82(3.65-3.99)	4.98(4.7-5.28)	4.22(4.07-4.36)
Myocardial infarction	1.07(1.01-1.12)	1.3(1.21-1.4)	1.13(1.08-1.18)
Congestive heart failure	1.33(1.29-1.37)	1.28(1.24-1.32)	1.31(1.28-1.34)
Cerebrovascular disease	1.43(1.37-1.5)	1.57(1.5-1.65)	1.49(1.45-1.55)
Dementia	2.13(2.05-2.22)	1.91(1.84-1.98)	2.01(1.96-2.07)
T2DM	0.89(0.87-0.92)	0.94(0.91-0.97)	0.92(0.9-0.94)
Hemiplegia or paraplegia	1.88(1.69-2.1)	1.99(1.73-2.28)	1.93(1.77-2.11)
Renal disease	1.14(1.1-1.17)	1.2(1.16-1.24)	1.16(1.14-1.19)
Cancer	2.1(2.02-2.18)	2.19(2.07-2.3)	2.12(2.06-2.19)
Moderate/severe liver disease	3.15(2.89-3.44)	2.95(2.57-3.38)	3.12(2.9-3.36)
Metastatic cancer	5.71(5.47-5.96)	6.62(6.21-7.05)	5.98(5.77-6.19)
AIDS	1.18(1.03-1.36)	NS	1.23(1.09-1.4)
Asthma	0.87(0.82-0.92)	0.60(0.56-0.63)	0.72(0.70-0.74)
Non-Invasive Mechanical ventilation	2.93(2.75-3.13)	2.71(2.52-2.91)	2.83(2.7-2.97)
Invasive Mechanical ventilation	8.11(7.67-8.57)	8.88(8.22-9.59)	8.37(8-8.76)
2016	1	1	1
2017	0.97(0.94-1.01)	0.97(0.93-1.01)	0.97(0.94-1)
2018	0.93(0.9-0.97)	0.93(0.89-0.97)	0.93(0.91-0.96)
2019	0.87(0.84-0.9)	0.86(0.83-0.9)	0.87(0.84-0.89)
Women	NA	NA	1
Men	NA	NA	1.08(1.06-1.1)

T2DM: type 2 diabetes mellitus; AIDS: acquired immune deficiency syndrome; NA: not applicable. NS: not significant. The absence of the condition or procedure is used as the reference category.
