# Supplementary figures and images for: Community-Acquired Pneumonia among Patients with COPD in Spain from 2016 to 2019. Cohort Study Assessing Sex Differences in the Incidence and Outcomes Using Hospital Discharge Data
Source: J Clin Med. 2021 Oct 23;10(21):4889. doi: 10.3390/jcm10214889 (PMC8584564; doi:10.3390/jcm10214889)

Figure S1. Flow chart of patient's selection and hospital outcome according COPD status and sex

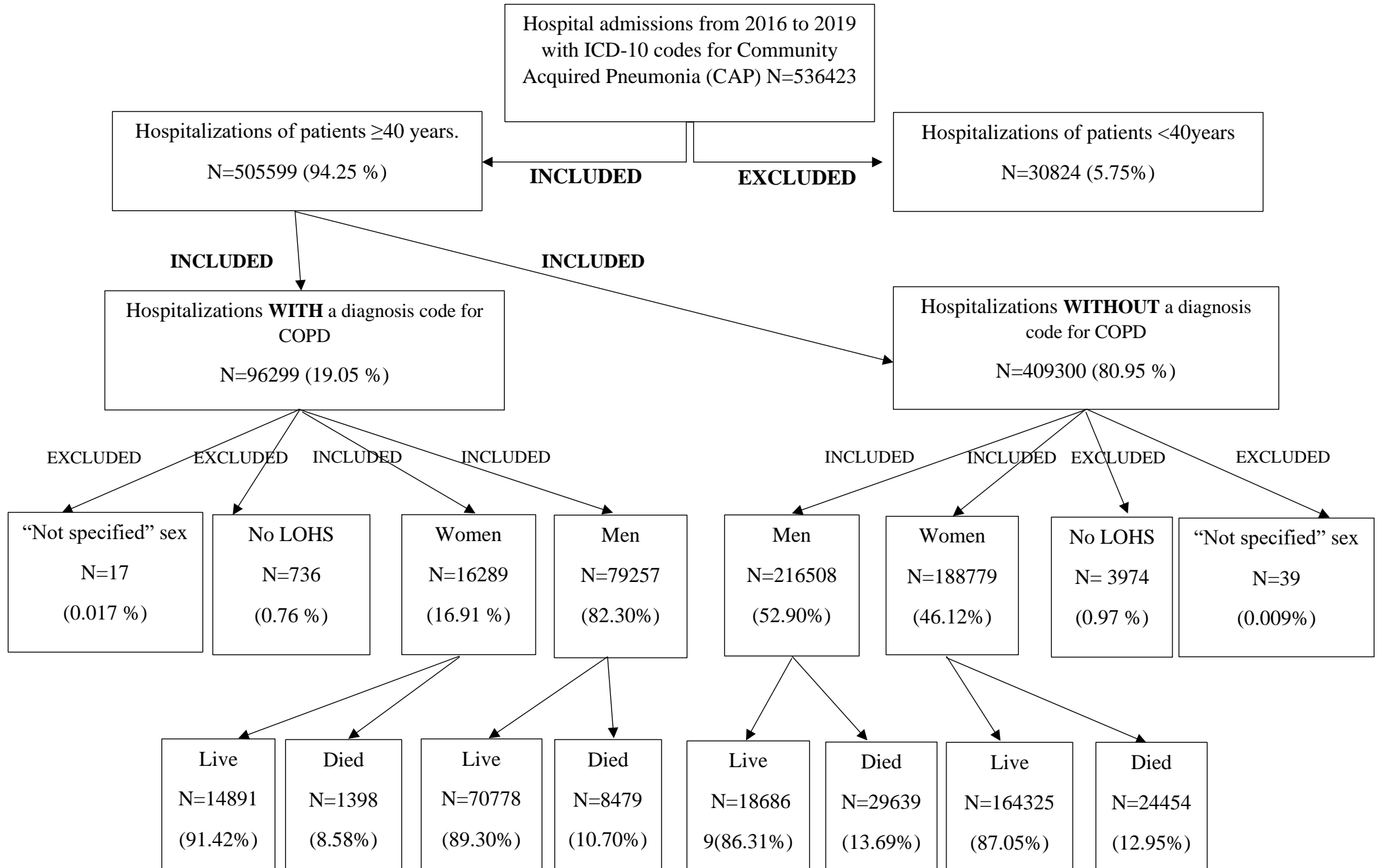

Supplement: Supplementary file 1 [file jcm-10-04889-s001.zip › FigureS1.pdf]
